# Coronavirus Disease 2019 (COVID-19) Complicated by Acute Respiratory Distress Syndrome: An Internist’s Perspective

**DOI:** 10.7759/cureus.7482

**Published:** 2020-03-31

**Authors:** Taha Ahmed, Ronak J Shah, Shab E Gul Rahim, Monica Flores, Amy O'Linn

**Affiliations:** 1 Internal Medicine, Cleveland Clinic Foundation, Cleveland, USA; 2 Internal Medicine, Cleveland Clinic - Fairview Hospital, Cleveland, USA; 3 Hospital Medicine, Cleveland Clinic - Fairview Hospital, Cleveland, USA

**Keywords:** covid-19, coronavirus disease 2019, acute respiratory distress syndrome, pandemic, nebulizer, corticosteroids, ards

## Abstract

A pandemic outbreak of a novel coronavirus disease (COVID-19) that began in Wuhan, China, in December 2019 has spread rapidly to multiple countries. In the United States, the first confirmed case was reported on January 20, 2020, and since then, the number of cases is rising exponentially on a daily basis. We report a case of COVID-19 infection that presented with symptoms suggestive of pneumonia. Due to the major backlog with an immense number of pending tests, it took 48 hours for the result to come back positive, while the patient went into acute respiratory distress syndrome. We provide an internist’s perspective of the difficulties encountered in terms of the available management options, as the patient progressively deteriorated on the regular medical floor prompting transfer to the intensive care unit.

## Introduction

Coronavirus disease (COVID-19) was first detected in December 2019 in China and is spreading at an exceptional pace around the world. On March 11, 2020, the World Health Organization (WHO) declared it as a global pandemic, with more than 353,000 cases reported to this date worldwide. Although cases were initially reported to be associated with exposure to the seafood market in Wuhan, China, current epidemiologic data indicate that person to person transmission of COVID-19 is rapidly occurring [1,2]. Herein, we present a challenging case of COVID-19 that tested positive 48 hours after initial testing due to major backlog on lab testing, and the management options at our hand to prevent the rapid deterioration of this fatal respiratory infection in the meantime.

## Case presentation

An 80-year-old male with a history of coronary artery disease post bypass grafting, paroxysmal atrial fibrillation (AF) on warfarin for stroke prophylaxis, acid reflux, hypertension, and chronic kidney disease presented to our emergency department with the chief complaint of fatigue, confusion, and dry cough for the last two weeks. The patient stated that the symptoms started while he was on vacation in Atlanta and he returned two days ago. The cough was described as dry with occasional greenish sputum associated with shortness of breath, sore throat, and chills but no fevers. He denied any sick contacts, hiking, backpacking, or exposure to bats. He reported to be a chronic smoker with a 40 pack-year smoking history and quit smoking a few years ago and drank alcohol socially.

**Figure 1:**
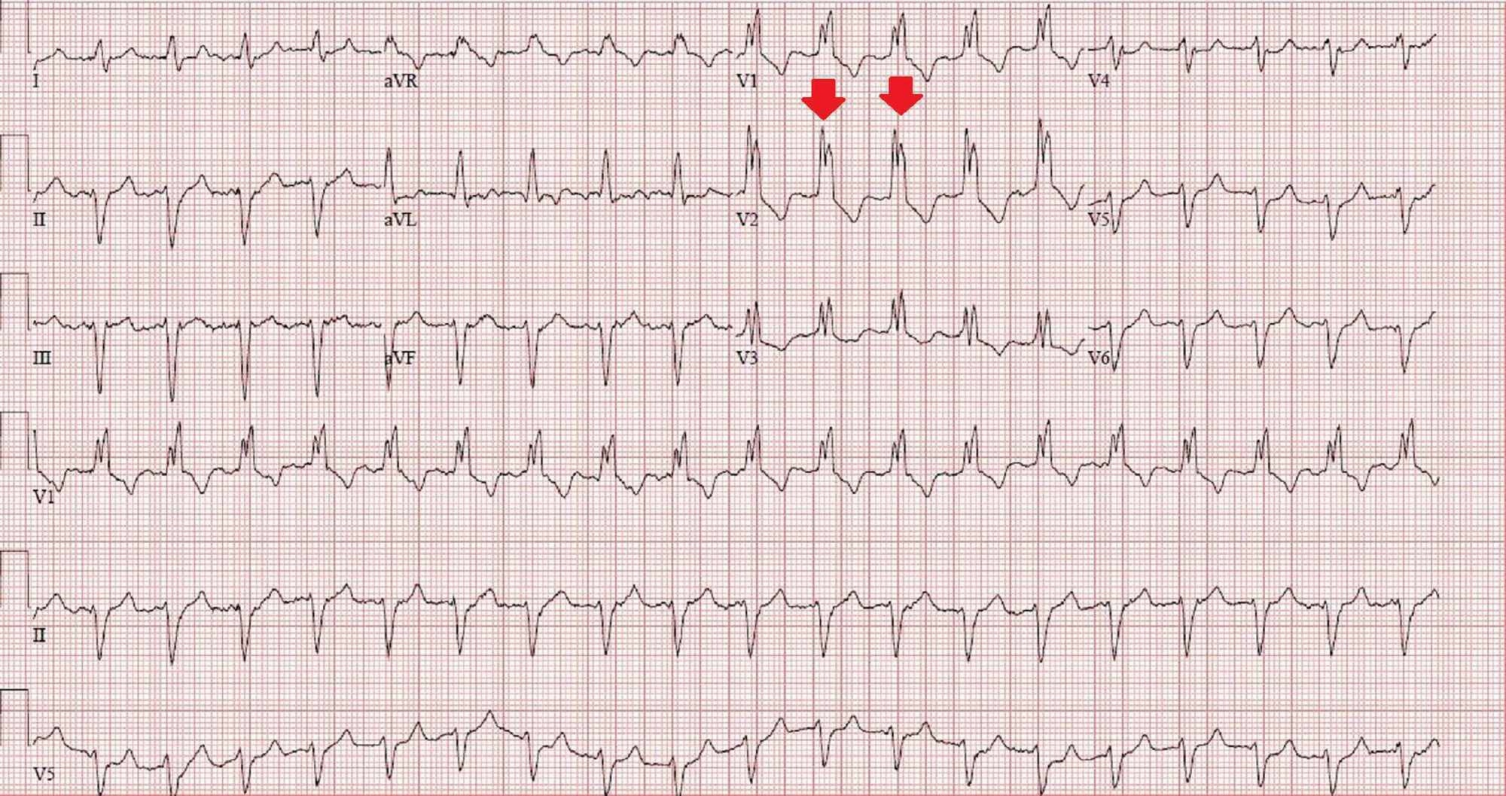
Electrocardiogram showing atrial fibrillation with complete right bundle branch block

On presentation, the patient was alert and oriented with no apparent confusion, and diffusely decreased breath sounds with occasional rhonchi were heard on right upper lung auscultation with irregularly irregular rhythm on cardiac auscultation. Vital signs revealed a heart rate (HR) of 110 beats per minute (BPM), a respiratory rate of 18 breaths per minute, a blood pressure of 102/45 mmHg, and an oxygen saturation of 90% on room air. Electrocardiogram showed AF with a complete right bundle branch block (Figure 1).

**Figure 2:**
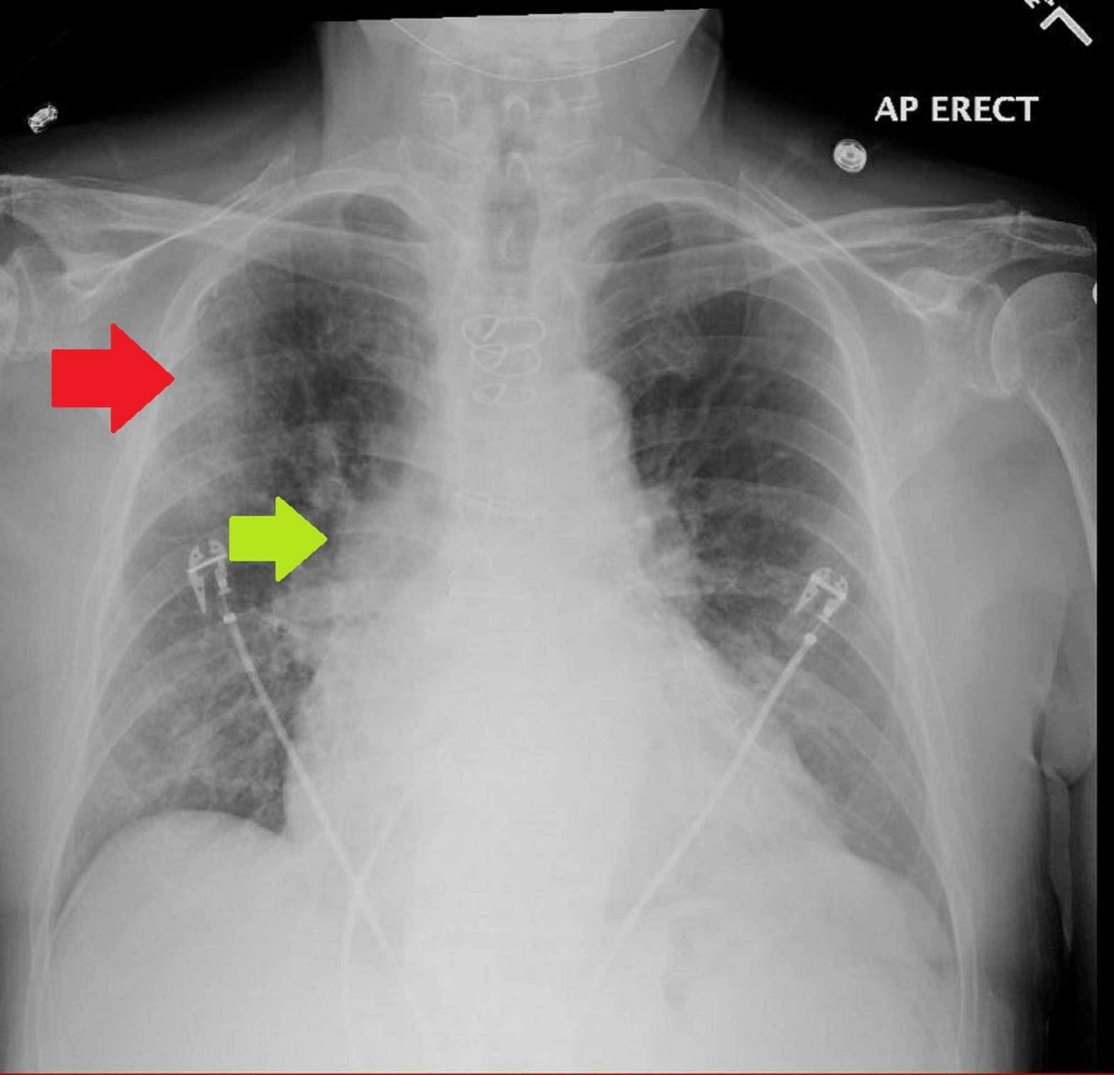
Chest X-ray on presentation showing hazy right upper lobe opacity (red arrow) with increased prominence of right hilum indicating adenopathy or inflammatory changes (green arrow)

The complete blood count was unremarkable, and metabolic profile revealed an elevated creatinine of 1.71 mg/dL (normal: 0.73-1.22 mg/dL) and magnesium level of 1 mg/dL (normal: 1.7-2.3 mg/dL). International normalized ratio was 2.8 on warfarin, brain natriuretic peptide was 1,246 pg/mL (normal: less than 450 pg/mL), and troponin T was normal. Chest X-ray showed new hazy right upper lobe lateral opacity concerning for an infiltrate with the increased prominence of right hilum indicating adenopathy or bronchiectatic inflammatory changes (Figure 2).

Influenza screen was negative; nasopharyngeal swab for coronavirus 19 testing was sent, blood cultures were drawn, and the patient was commenced on intravenous doxycycline and ceftriaxone before transfer to our service on the regular medical floor (RMF).

On the RMF, the patient was initially managed as community-acquired pneumonia with a COVID-19 rule out status. All protective measures and precautions for suspected COVID-19 infection were taken, and the patient was placed in isolation. Intravenous antibiotics were continued, and bacterial pneumonia workup and procalcitonin were sent.

Overnight, the patient became tachycardiac (HR, 130 BPM) and spiked a fever of 101.5°C, with physical examination revealing worsening bilateral wheezing and intermittent delirium. Sepsis lactate was 1 mmol/L (normal: 0.5-2 mmol/L) and C-reactive protein was 6.1 mg/dL (normal: 0-1 mg/dL). Bacterial pneumonia workup came back negative and COVID testing was still pending amid severe backlog. AF with a rapid ventricular response (RVR) was deemed to be due to acute illness, and home metoprolol dose was increased. Instructions were given to avoid nebulizer treatments and oral/parenteral steroids due to the pending COVID 19 status.

On the morning of day 2 of admission, the patient became progressively hypoxic and his oxygen demands acutely increased, with worsening diffuse wheezing on examination. Attempts were made to relax the airway with ipratropium and budesonide inhalers, with minimal effect. Eventually, the rapid response team was called in the afternoon of day 2 for persistent hypoxia and AF with RVR. Arterial blood gas revealed acute hypoxic respiratory failure with a P:F of 140.

**Figure 3:**
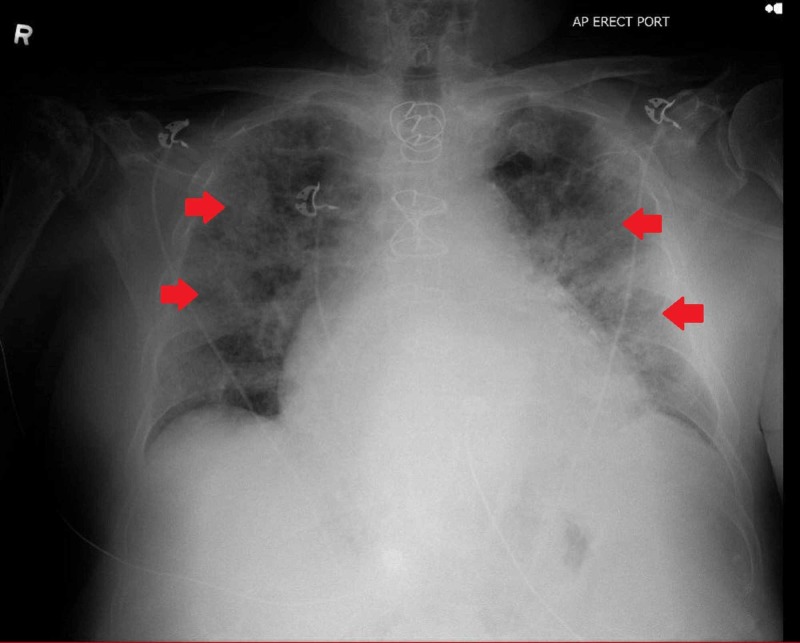
Chest X-ray on day 2 of admission revealing worsening infiltrates in bilateral lung zones

Chest X-ray this time around showed worsening infiltrates bilaterally (Figure 3).

**Figure 4:**
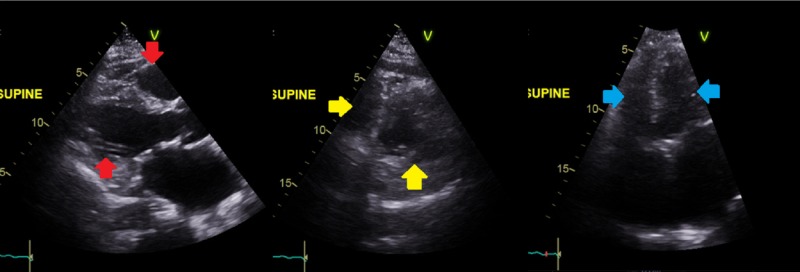
Transthoracic echocardiogram showing normal biventricular size and function in parasternal long axis (red arrows), parasternal short axis (yellow arrows), and apical four chamber (blue arrows) views

Transthoracic echocardiogram showed normal biventricular size and function (Figure 4).

Intravenous steroids were administered, and the patient was transferred to the intensive care unit (ICU). En route to the ICU, the patient’s COVID 19 test came back positive, approximately 48 hours after it was ordered. The patient was intubated and put on mechanical ventilation in the ICU under airborne precautions, avoiding non-invasive ventilation and commenced on hydroxychloroquine and azithromycin, as an initial step with an aim to add on other proposed therapies (remdesivir, tocilizumab, lopinavir-ritonavir). The patient currently remains in the ICU, critically ill with a poor prognosis.

## Discussion

Coronavirus (CoV) is a large family of positive-sense, single-stranded RNA viruses that belong to the Nidovirales order. The order includes Roniviridae, Arteriviridae, and Coronaviridae families [3]. The Coronaviridae family is subdivided into Torovirinae and Coronavirinae families [3]. Coronavirinae subfamily is further subclassified into alpha-, gamma-, and delta-CoVs [4]. Their viral RNA genome ranges from 26 to 32 kilobases in length. Usually found in mammals and birds, human pathogenic subtypes are associated with mild clinical symptoms. However, severe acute respiratory syndrome-related coronavirus (SARS-CoV) and Middle East respiratory syndrome coronavirus (MERS-CoV) are two notable exceptions [4,5].

In the past 20 years, two CoV epidemics have occurred. SARS-CoV provoked a large-scale epidemic beginning in China in 2002 and involving two dozen countries with approximately 8,000 cases and 800 deaths, and the MERS-CoV that began in Saudi Arabia in 2012 and has approximately 2,500 cases and 800 deaths and still causes sporadic cases. In a timeline that reaches to the current day, an epidemic of cases with unexplained lower respiratory tract infections detected in Wuhan, a large metropolitan area in China’s Hubei province, was first reported to the WHO county office in China on December 31, 2019. As the cases grew in number, the Chinese Center for disease control (CDC) organized an intensive outbreak investigation program. The etiology of this illness is now attributed to a novel CoV, COVID-19. On February 11, 2020, the WHO Director-General, Dr. Tedron Ghebreyesus, announced that the disease caused by this new CoV was COVID-19 an acronym of “coronavirus disease 2019” [6]. This new virus is very contagious and is spreading quickly globally and has been labeled as a global pandemic earlier this month by WHO.

As of March 28, 2020, the WHO has confirmed 683,694 confirmed cases of COVID-19 infection. A total of 32,155 (4.70%) cases have succumbed to the virus. The disease has affected more than 189 nations around the globe. As per the CDC, the United States has the most number of cases (>123,000) followed by Italy (>92,000) and China (>81,000) cases.

Because the first case of the COVID-19 disease was linked to direct exposure to the Huanan Seafood Wholesale Market of Wuhan, animal to human transmission was presumed as the main mechanism. However, subsequent cases were not associated with this exposure mechanism. Therefore, it was concluded that the virus could also be spread from human to human, and symptomatic people are the most frequent source of COVID-19 spread.

Currently, our understanding of the clinical spectrum of COVID-19 disease is very limited [7]. Complications including severe pneumonia, acute respiratory distress syndrome (ARDS), and cardiac injury, including fatal outcomes, have been reported in China [8,9]. Clinical and epidemiological data from the Chinese CDC report regarding 72,314 case records (confirmed, suspected, diagnosed, and asymptomatic cases) were shared in the Journal of the American Medical Association (JAMA), providing vital epidemiologic information in the Chinese outbreak. There were 62% confirmed cases with a case-fatality ratio of 2.3%. Of note, the fatal cases were primarily elderly (>80 years, 15% and 70 to 79 years, 8%). Approximately half of the critical patients affected by pre-existing comorbidities such as cardiovascular disease, diabetes, chronic respiratory disease, and oncologic disease, died. The authors classified the disease as mild, moderate, and severe disease (severe pneumonia, ARDS, septic shock) [10]. Our case manifested as a severe case of COVID-19 disease.

There is no specific antiviral medication to date for COVID-19, and no vaccine is currently available. Symptomatic treatment is currently the mode of practice and treatment primarily depends upon the severity of illness. Our patient rapidly deteriorated within 48 hours of presentation requiring mechanical ventilation. During those 48 hours on the RMF, it was a challenging encounter regarding the management. The patient likely had worsening of his underlying chronic obstructive pulmonary disease due to the extensive smoking history. Nebulizer treatment was avoided, as the risk of infection transmission via droplet nuclei and aerosols increases during nebulizer treatments because of the potential to generate a high volume of respiratory aerosols that may be propelled over a longer distance than that involved in the natural dispersion pattern [11]. Furthermore, the larger aerosol particles (1-5 µm) may stimulate both the patients and bystanders to cough, thus increasing the risk of the disease spread. Nebulizer therapy in patients with pandemic COVID-19 infection has the potential to transmit potentially viable COVID-19 to susceptible bystander hosts.

In our patient, metered dose inhalers were utilized but with minimal benefit. Corticosteroids (oral/parenteral) were avoided as per the WHO guidance and published recommendations [12]. However, when the patient went into ARDS, it became prudent to administer parenteral corticosteroids as treatment with parenteral steroids has shown to decrease the risk of death in patients with COVID-19 disease and ARDS [13]. The patient became gradually more hypoxic and eventually went into moderate-to-severe ARDS, requiring invasive ventilator support.

## Conclusions

We report a case of COVID-19 infection deteriorating to moderate-to-severe ARDS in a matter of 48 hours. As an Internist, we provide a perspective of the scarcity of the available treatment modalities in our arsenal. The exponential spread of the disease warrants intense surveillance and isolation protocols to be implemented to prevent further spread. Health care workers should have a thorough understanding of the presentation of the disease, workup, and management of these patients, as well as necessary precautions, to avoid contact and spread of the disease.
